# Thoracoscopic resection of a paraaortic bronchogenic cyst

**DOI:** 10.1186/1749-8090-5-82

**Published:** 2010-10-15

**Authors:** Baldassare Mondello, Salvatore Lentini, Dario Familiari, Pietro Barresi, Francesco Monaco, Michele Sibilio, Annunziata La Rocca, Vincenzo Micali, Ignazio Eduardo Acri, Mario Barone, Maurizio Monaco

**Affiliations:** 1 Thoracic Surgery Unit, Cardiovascular and Thoracic Department, Policlinic University Hospital, University of Messina, Italy

## Abstract

Bronchogenic mediastinal cysts (BMC) represent 18% of primitive mediastinal tumors and the most frequent cystic lesions in this area. Nowadays, BMC are usually treated by VATS. However, the presence of major adhesions to vital structures is often considered as an unfavourable condition for thoracoscopic treatment. The authors report the thoracoscopic treatment of a BMC having dense adhesions to the aortic arch. Diagnosis and surgical treatment is described. Review of the literature and surgical options on this topic are discussed.

## Background

Bronchogenic mediastinal cysts (BMC) represent 18% of primitive mediastinal tumors, and are the most frequent cystic lesions in this anatomic region [1,2]. Surgical resection is recommended. Video assisted thoracic surgery (VATS) has been reported for the resection of these lesions. However, the presence of major adhesions to vital structures is considered by some authors as an unfavorable condition for BMC treatment by VATS. We report a surgical approach by VATS for a BMC with adhesion to the aortic arch. Diagnosis and treatment of the specific case is reported with literature review and therapeutic options.

## Case Presentation

**Figure 1:**
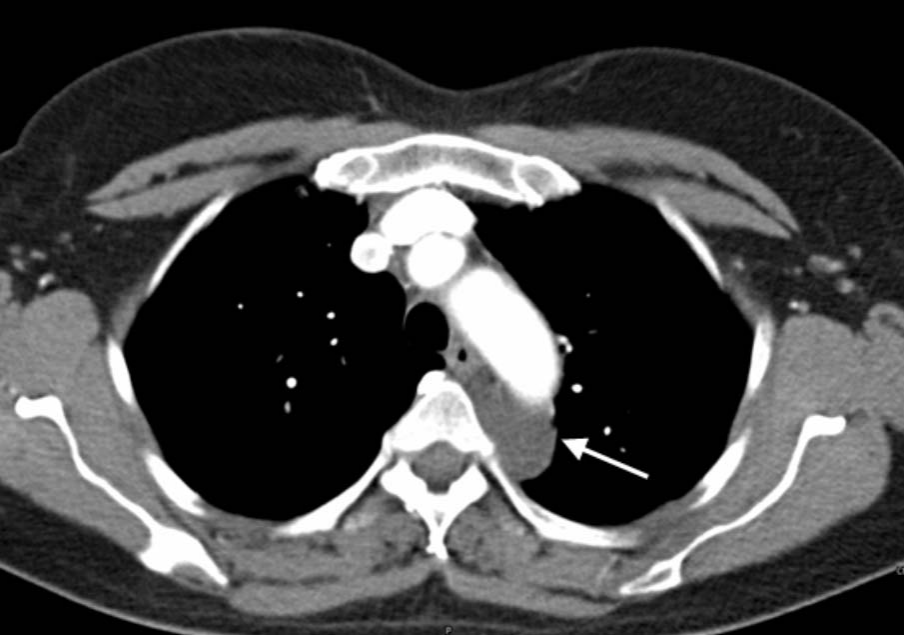
**CT scan showing a cystic lesion**. CT scan showing a cystic lesion (white arrow) located between the aortic arch and the thoracic spine.

**Figure 2:**
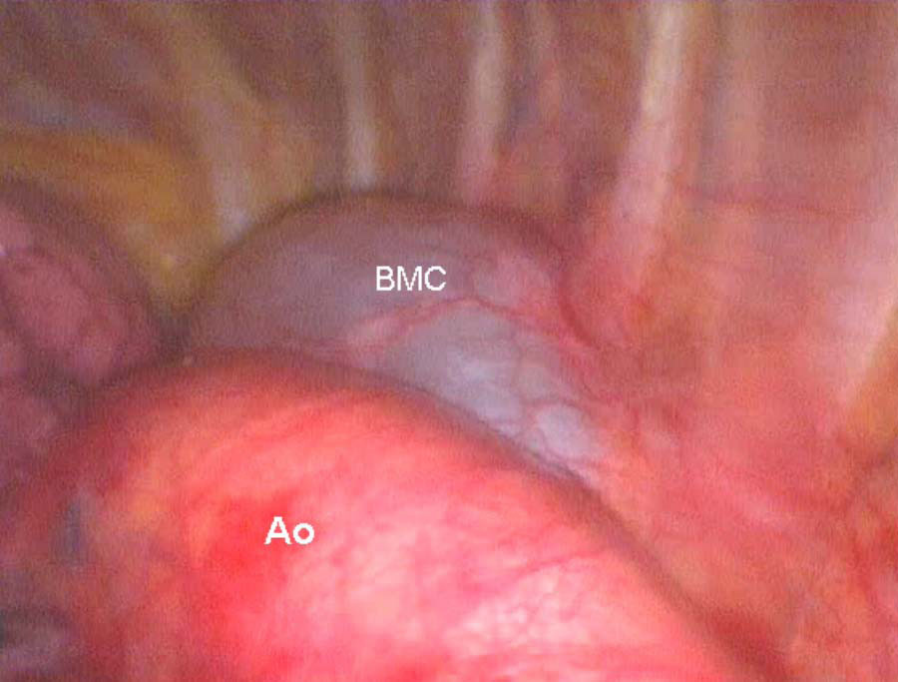
**Thoracoscopic finding**. Thoracoscopic finding: large cystic lesion with adhesion to the aortic arch. BMC: Bronchogenic mediastinal cyst. Ao: Aorta

**Figure 3:**
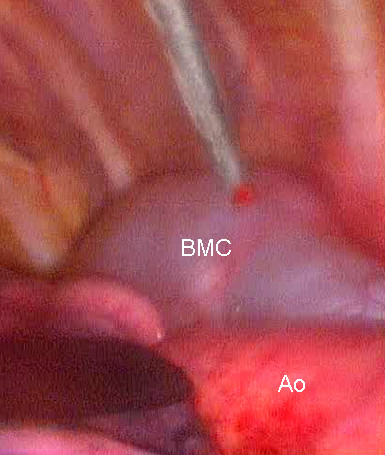
**Intraoperative steps**. Intraoperative steps: Needle aspiration of cystic fluid. BMC: Bronchogenic mediastinal cyst. Ao: Aorta

**Figure 4:**
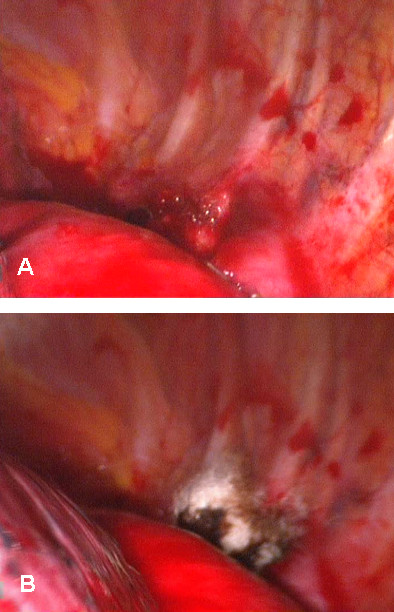
**Final surgical steps**. Final surgical steps showing: a) The cyst has been completely removed. b) Diathermy burning of the parietal pleura where cyst adhesions were present.

A 50 year old asymptomatic woman was referred to our out-patient clinic following occasional detection of a mediastinal mass. On routine chest x-ray performed before orthopaedic surgery, the suspicion arose of a mediastinal mass. A computed tomography (CT) scan showed a cystic mass in the posterior mediastinum between the aortic arch and the vertebral bodies (Figure 1). The cyst extended from the 3^rd ^thoracic vertebral body to the tracheal carina plane, with a length of 4 cm and a transversal diameter of 2.5 cm. The lesion appeared cystic with a well defined capsule and lacking enhancement after intravenous contrast injection. Surgical treatment was decided upon. Preoperative bronchoscopy excluded any communication between the cyst and the tracheobronchial tree. After double lumen intubation, the patient was placed in a right lateral position on the operating table. Three trocars were used: one on the fifth intercostal space along the anterior axillary line; one on the fifth intercostal space along the posterior axillary line; and the last one on the 7^th ^intercostal space along the midaxillary line. The cyst was visualized by thoracoscopy, appearing with a major adhesion on the distal portion of the aortic arch (Figure 2). To facilitate surgical dissection of the cystic lesion from the aorta, fluid aspiration was performed (Figure 3). Once the cyst was empty, complete resection from the adherent aorta was easily completed (Figure 4a). However, despite total lesion excision, we completed the surgical procedure by passing the electrocautery on the pleural area where the cyst was adherent (Figure 4b). The procedure was completed with insertion of a chest tube. Histology examination confirmed the diagnosis of benign bronchogenic cyst with the typical feature of a ciliated columnar epithelial lining. The postoperative (PO) course was uneventful and the patient was discharged home on the 5^th ^PO day. At 12 months follow-up the patient remains well with no recurrence on control CT scan.

## Discussion

Bronchogenic mediastinal cysts (BMC) are a rare pathology, accounting for 18% of all primitive mediastinal tumors and represent the most frequent cystic lesions in this anatomic region [1-3].

They represent congenital malformations arising from an abnormal division of the tracheobronchial tree. In relation to the time of separation from the main tracheobronchial tree, the cysts may localize into the lung parenchyma or in the mediastinum, with percentages of 33% and 66%, respectively [4-6].

They are usually unilocular, rarely multilocular. Their wall is represented by a ciliated columnar epithelium lining, cartilage structure and occasionally may contain a mucinoid filling. BMC are usually asymptomatic, and often casually diagnosed. When present, symptoms are usually related to the area of occurrence and include chest pain, cough, dyspnoea, dysphagia, or emoptysis [7,8].

Complications may occur, including infection, emoptysis, trachea or superior vena cava compression, intracystic haemorrhage, rupture, bronchial fistula, pneumothorax, and malignant changes, which have all been reported [9-13]. For this reason once the diagnosis of MBC is done, even if asymptomatic, surgical resection may be recommended. Complete resection represents the therapeutic gold standard, minimizing the recurrence incidence. Standard treatment has been usually by thoracothomy [14]. VATS treatment gradually became the first option also for BMC [15-17]. However, the presence of BMC with major adhesion to vital structures has been considered as an unfavorable condition for VATS treatment [14]. In our case, we treated the BMC by VATS despite the important adhesion on the aortic arch. We believe that cautious dissection of the cystic lesion after needle aspiration may prove useful in this setting. Intraoperative cyst aspiration may help in the handling of the lesion, reducing the risk of rupture. The advantage of thoracoscopy treatment is evidenced by reduced discomfort for the patient. The decreased postoperative pain is a result of the lack of intercostal incisions. Hospital stay and chest tube duration are lower as compared to open thoracothomy [17]. A relevant reason for conversion to open surgery would be major pleural adhesions [9,14]. Aspiration of the cyst fluid has been recommended with the aim of facilitating cyst preparation [18]. We used cyst aspiration during surgical dissection in order to better separate the cystic structure from the underlying aorta. When the cyst has adhesion to vital structures, surgical removal may be somewhat hazardous and incomplete removal may predispose to recurrence. In those cases, the use of diathermy may be useful in completing the surgical excision [8,19]. In our case, even after total excision, we completed the surgical approach with the use of electrocautery to the area of cyst attachment. Late recurrences have been reported, even after 25 years [20]. In any case, incomplete cyst excision has been reported not only for VATS but also for open surgery [5,8-10,14]. Cyst rupture during preparation does not prevent the procedure being completed by VATS [21]. Accurate preoperative imaging studies have been recommended to better plan the operative strategy. Computed tomography (CT) scan and magnetic resonance imaging (MRI) are considered the best methodologies for preoperative diagnosis, with 100% reported accuracy for MRI [8]. Transesophageal ultrasonography may be useful, especially if an esophageal duplication cyst with communication to the esophagus is suspected [22].

Transthoracic and transbronchial needle aspiration has been used both diagnostically and therapeutically [23-25]. However, complete resection still represents the gold standard treatment.

## Conclusion

In conclusion, we believe a bronchogenic cyst should be treated by complete surgical resection. As previously reported by others, in relation to the advantages of the minimally invasive approach, we believe VATS treatment should be considered as the gold standard therapy. This approach may prove useful also in cases where there are adhesions to vital structures such as the aortic arch.

## Competing interests

The authors declare that they have not competing interests.

## Authors' contributions

All authors: 1. have made substantial contributions to conception and design, or acquisition of data, or analysis and interpretation of data; 2. have been involved in drafting the manuscript or revisiting it critically for important intellectual content; 3. have given final approval of the version to be published.

## Consent

Written informed consent was obtained from the patient for publication of this case report and accompanying images. A copy of the written consent is available for review by the Editor in chief of this journal.

## References

[B1] Wychulis AR Payne WS Clagett OT Woolner LB Surgical treatment of mediastinal tumors. A 40 year experience J Thorac Cardiovasc Surg 1971 62 379 92 4331304

[B2] Bolton JW Shahian DM Asymptomatic bronchogenic cysts: what is the best treatment? Ann Thorac Surg 1992 53 1134 7 10.1016/0003-4975(92)90412-W 1596146

[B3] Takeda S Miyoshi S Minami M Ohta M Masaoka A Matsuda H Clinical spectrum of mediastinal cysts Chest 2003 124 125 32 10.1378/chest.124.1.125 12853514

[B4] O'Rahilly R Müller F Respiratory and alimentary relations in staged human embryos. New embryological data and congenital anomalies Ann Otol Rhinol Laryngol 1984 93 421 9 6497234 10.1177/000348948409300501

[B5] St-Georges R Deslauriers J Duranceau A Vaillancourt R Deschamps C Beauchamp G Pagé A Brisson J Clinical spectrum of bronchogenic cysts of the mediastinum and lung in the adult Ann Thorac Surg 1991 52 6 13 10.1016/0003-4975(91)91409-O 2069465

[B6] McAdams HP Kirejczyk Rosado de Christenson ML Matsumoto S Bronchogenic cyst: imaging features with clinical and histopathologic correlation Radiology 2000 56 441 6 10.1148/radiology.217.2.r00nv19441 11058643

[B7] Patel SR Meeker DP Biscotti CV Kirby TJ Rice TW Presentation and management of bronchogenic cysts in the adult Chest 1994 106 79 85 10.1378/chest.106.1.79 8020324

[B8] Kanemitsu Y Nakayama H Asamura H Kondo H Tsuchiya R Naruke T Clinical features and management of bronchogenic cysts: report of 17 cases Surg Today 1999 29 1201 5 10.1007/BF02482273 10552342

[B9] Ribet ME Copin MC Gosselin B Bronchogenic cysts of the mediastinum J Thorac Cardiovasc Surg 1995 109 1003 10 10.1016/S0022-5223(95)70327-6 7739231

[B10] Aktogu S Yuncu G Halilcolar H Ermete S Budunelli T Bronchogenic cysts: clinicopathological presentation and treatment Eur Respir J 1996 9 2017 21 10.1183/09031936.96.09102017 8902460

[B11] Miralles Lozano F Gonzalez-Maritez B Luna More S Valencia Rodriguez A Carcinoma arising in a calcified bronchogenic cyst Respiration 1981 42 135 7 7313333 10.1159/000194417

[B12] Endo C Imai T Nakagawa H Ebina A Kaimori M Bronchioloalveolar carcinoma arising in bronchogenic cyst Ann Thorac Surg 2000 69 933 5 10.1016/S0003-4975(99)01402-2 10750790

[B13] De Perrot M Pache JC Spilipoulos A Carcinoma arising in congenital lung cyst Thorac Cardiovasc Surg 2001 49 184 5 10.1055/s-2001-14284 11432479

[B14] Martinoid E Pons F Azorin J Mouroux J Dahan M Faillon JM Dujon A Lajos PS Riquet M Jancovici R Thoracoscopi excision of mediastinal bronchogenic cysts: results in 20 cases Ann Thorac Surg 2000 69 1525 8 10.1016/S0003-4975(99)01438-1 10881835

[B15] Mouroux J Bourgeon A Benchimol D Bernard JL Chazai M Padovani B Richelme H Bronchogenic cysts of the esophagus. Classic surgery or videosurgery? Chirurgie 1991 117 564 8 1842953

[B16] Weber T Roth CT Bshay M Herrmann P Stein R Schmid RA Videoassisted thoracoscopic surgery of mediastinal bronchogenic cysts in adults: a single-center experience Ann Thorac Surg 2004 78 987 91 10.1016/j.athoracsur.2004.03.092 15337033

[B17] Tölg C Abelin K Laudenbach V de Heaulme O Dorgeret S Lipsyc ES Aigrain Y de Lagausie P Open vs Thoracoscopic surgical management of bronchogenic cysts Surg Endosc 2005 19 77 80 10.1007/s00464-003-9328-x 15549633

[B18] Hazelrigg SR Landreneau RJ Mack MJ Acuff TE Thoracoscopic resection of mediastinal cysts Ann Thorac Surg 1993 56 659 60 10.1016/0003-4975(93)90944-D 8379765

[B19] Lewis RJ Caccavale RJ Sisler GE Imaged thoracoscopic surgery: a new thoracic technique for resection of mediastinal cysts Ann Thorac Surg 1992 53 318 20 10.1016/0003-4975(92)91340-F 1731675

[B20] Read CA Moront M Carangelo R Holt RW Richardson M Recurrent bronchogenic cysts. An argument for complete surgical excision Arch Surg 1991 126 1306 8 1929835 10.1001/archsurg.1991.01410340148022

[B21] De Giacomo T Diso D Anile M Venuta F Rolla M Ricella C Coloni GF Thoracoscopic resection of mediastinal bronchogenic cysts in adults Eur J Cardioth Surg 2009 36 357 59 10.1016/j.ejcts.2009.03.041 19411178

[B22] Van Dam J Rice TW Sivak MV Jr Endoscopic ultrasonography and endoscopically giuded needle aspiration for the diagnosis of upper gastrointestinal tract foregut cysts Am J Gastroeneterol 1992 87 762 5 1590317

[B23] McDougall JC Fromme GA Transcarinal aspiration of a mediastinal cyst to facilitate anesthetic management Chest 1990 97 6 1490 2 10.1378/chest.97.6.1490 2347240

[B24] Kuhlman JE Fishman EK Wang KP Zerhouni EA Siegelman SS Mediastinal cyst: diagnosis by CT and needle aspiration AJR Am J Roentgenol 1988 150 1 75 8 3257135 10.2214/ajr.150.1.75

[B25] Galluccio G Lucantoni G Mediastinal bronchogenic cyst's recurrence treated with EBUS-FNA with a long-term follow-up Eur J Cardiothorac Surg 2006 29 627 9 10.1016/j.ejcts.2005.12.052 16476541

